# Case of Tinea

**Published:** 1803-12-01

**Authors:** M. Baysillance

**Affiliations:** Member of the Society of Medical Instruction, at Paris


### Case of Tinea; by M. Baysillance, M. D. *Member of the Society of Medical Instruction, at Paris.*

A. B. aged eighteen years, of a lymphatico-nervous temperament, born of healthy parents, having many brothers who enjoyed good health, experienced no complaint in her early years, which had any relation to the one in question. At five years of age, having frequented children affected with tinea, and using the same comb, small buttons, at first few in number, appeared in different points of the head; in the beginning, slight itching only was experienced, but this considerably increased, as well as the pimples, so that these last now covered the entire hairy scalp, forming scabs which frequently dried, fell, and returned again; a considerable quantity of insupportably fœtid matter constantly oozed from the surface. This person underwent different kinds of treatment, the most exact and regular of which was when she was nine years old. She was purged every eight days. Soap suds and volatile alkali were applied topically, and latterly water-cresses boiled in hog's lard, was used; this treatment was continued three months; the scabs disappeared, but soon returned with the same intensity. On the application of any greasy substance the scabs disappeared. At the age of fourteen, the menses appeared, at first in sufficient quantity, but afterwards with great irregularity, without however occasioning any change in the state of health; at this period the scabs disappeared, and the girl was supposed to be radically cured; at the end of a month, however, the complaint returned, but less severely; the pustules and scabs came and went frequently. When the author saw the patient, she was in the following state: The body was much emaciated, the health was however good; the lymphatic glands of the neck, arm pits, &c. did not seem affected. The head, cleared of the scabs, &c. presented a considerably red appearance; viewed through a lens, there was remarkable, 1. Small round ulcers at the root of each hair, from which oozed a yellowish matter. 2. The hairs which appeared to float in the centre of these ulcers, were easily withdrawn without creating any pain, their root was covered with a matter thicker than common pus. The treatment* was commenced by some pur-

* This method had been previously employed by M. Dubois, in the Hospital of the Medical School, called Ecole de Perfectionnement, of which he is chief Surgeon.

purgatives composed of 24 grains of jalap and some sugar, and which was renewed every eight days during the cure. After cutting the hair as closely as possible, the head was rubbed with the following ointment, composed of linseed and juniper berries, reduced to a coarse powder, of each six ounces, thirty leaves of laurel bruised; the whole boiled in two pounds of lard. On the sixth of February, the patient was ordered to use the following medicine: Mild muriate of mercury, an ounce; fixed salt of tartar (tartarite of pot-ash) and ipecacuanha of each a drachm and a half, boiled in a pint of water till reduced to half; of this a large spoonful was taken night and morning. On the tenth, the mouth became sore, when the medicine was suspended. On the eleventh, it was resumed. On the twenty-ninth, in the dose of a tea spoonful in the twenty-four hours. On the fourth of March, the quantity was doubled. On the fifth, the head was clean, and the small pustules, already mentioned, only were perceivable. On this day, the author began to pull out the hairs with a dissecting forceps. On the twelfth, all the parts where the hair had been withdrawn, appeared perfectly well; here and there small tubercles remained in those places where the hair had been broken; on being opened and viewed through a lens, the disposition of the scabs was very perceptible. On the twenty-fourth, a blister was ordered to the left arm, and continued for three months; the work of pulling out the hairs was continued, and the pomatum already mentioned was changed for one made of fresh, red, rose leaves, in which a drachm and a half of common salt was mixed. The thirtieth every treatment was laid aside, except the blisters, which continued some time longer. The patient was purged twice.

The seat of tinea being, according to the author, in the corpus mucosum, adjoining the roots of the hair, he thinks there is no other way of combating the disease, than by causing their destruction. This is the opinion of many moderns, as well as the ancients. The very great pain attending this remedy, has given rise to many methods, none of which appear to the author to be preferable to the one he has mentioned. Tearing out the hair must be preferred in every case, except where the disease is complicated with caries of the bones of the head. The length of the operation may be objected to, but humanity sets this consideration aside. The author recommends the head to be constantly besmeared with the ointment during the extraction, and to extract only the hair of the diseased part; here they easily give give way; and lastly, to repeat the operation if the complaint returns.

We have reported this in the author's own words. The mode of treatment is the same as that practised by Professor Dubois, and in which we can find nothing new or remarkable, but the laudable patience of the author in pulling out the hair of the diseased part. We cannot see the use of the singular mercurial composition of which he makes use; and if the pulling out the roots of the hair in the diseased part, effects the cure, which we believe, what is the object of the ointment, unless it be intended to act as common grease? But above all, what is the view of blistering the arm, except it be to act as an issue, in which case as effectual and less troublesome means might be employed?

Humoral pathology is a doctrine very prevalent in the French school. The ideas of derivation, revulsion, metastasis of humours, &c. are found in the most modern practical books; diseases of the skin are treated with all the precautions connected with the belief of the affection being caused by humours, which, thrown back on the constitution, become dangerous. In the histories of most cases we are informed the person had some years before the itch, or some such affection, which was incautiously treated; hence we can understand the intention of purging mercurial preparations, issues, &c.

